# The Parasite Reduction Ratio (PRR) Assay Version 2: Standardized Assessment of *Plasmodium falciparum* Viability after Antimalarial Treatment In Vitro

**DOI:** 10.3390/ph16020163

**Published:** 2023-01-23

**Authors:** Annabelle Walz, Maëlle Duffey, Ghaith Aljayyoussi, Sibylle Sax, Didier Leroy, Dominique Besson, Jeremy N. Burrows, Mohammed H. Cherkaoui-Rbati, Nathalie Gobeau, Marie-Anne Westwood, Christoph Siethoff, Francisco-Javier Gamo, Pascal Mäser, Sergio Wittlin

**Affiliations:** 1 Swiss Tropical and Public Health Institute, 4123 Allschwil, Switzerland; 2 University of Basel, 4001 Basel, Switzerland; 3 Medicines for Malaria Venture, 1215 Geneva, Switzerland; 4 Liverpool School of Tropical Medicine, Centre for Drugs and Diagnostics, Liverpool L3 5QA, UK; 5 Swiss BioQuant, 4153 Reinach, Switzerland; 6 Global Health Medicines, GlaxoSmithKline I+D, 28760 Madrid, Spain

**Keywords:** malaria, *Plasmodium falciparum*, parasite viability, limiting dilution, parasite reduction ratio, PRR, PCT, lag phase, pharmacodynamics, E_max_

## Abstract

With artemisinin-resistant *Plasmodium falciparum* parasites emerging in Africa, the need for new antimalarial chemotypes is persistently high. The ideal pharmacodynamic parameters of a candidate drug are a rapid onset of action and a fast rate of parasite killing or clearance. To determine these parameters, it is essential to discriminate viable from nonviable parasites, which is complicated by the fact that viable parasites can be metabolically inactive, whilst dying parasites can still be metabolically active and morphologically unaffected. Standard growth inhibition assays, read out via microscopy or [^3^H] hypoxanthine incorporation, cannot reliably discriminate between viable and nonviable parasites. Conversely, the in vitro parasite reduction ratio (PRR) assay is able to measure viable parasites with high sensitivity. It provides valuable pharmacodynamic parameters, such as PRR, 99.9% parasite clearance time (PCT_99.9%_) and lag phase. Here we report the development of the PRR assay version 2 (V2), which comes with a shorter assay duration, optimized quality controls and an objective, automated analysis pipeline that systematically estimates PRR, PCT_99.9%_ and lag time and returns meaningful secondary parameters such as the maximal killing rate of a drug (E_max_) at the assayed concentration. These parameters can be fed directly into pharmacokinetic/pharmacodynamic models, hence aiding and standardizing lead selection, optimization, and dose prediction.

## 1. Introduction

The emergence of *Plasmodium falciparum* infections with delayed parasite clearance in response to artemisinin treatment first in Southeast Asia [1] and now also in Africa [2,3] poses a threat to current antimalarial treatments. Together with the rising annual malaria cases and deaths [4], this emphasizes the urgent need for new antimalarial combination medicines that overcome all existing resistance, and hence the need for novel candidate drugs to fill the drug development pipeline. A new candidate drug should, ideally, present a rapid onset of drug action and a high rate of parasite killing or clearance [5] in order to reduce clinical symptoms rapidly. To accurately determine these pharmacodynamic (PD) parameters, it is essential to discriminate between viable and nonviable parasites. However, in vitro, this is complicated by the fact that viable parasites can be metabolically inactive (e.g., dormant parasites), whilst dying or dead parasites might still be metabolically active and therefore detectable [6]. Furthermore, measurements of total parasitemia in vivo may fail to distinguish these parasite populations at all depending on the clearance rate of dead parasites [7,8,9]. Indeed, misclassification of viable parasites as nonviable (or vice versa) following treatment leads to inaccurate PD estimates and thus to an over- or underestimated drug activity.

The importance of parasite viability as a measure of drug activity was highlighted by a range of studies conducted in vitro [6,10], in mice [9], in human volunteers [8], and even in malaria patients [11]. These studies demonstrated that microscopy, metabolism-based assays, and even qPCR analysis provide inaccurate estimates of drug activity concerning parasite viability. Still, the use of parasite viability determination to investigate drug activity is scarce—likely because of the labor-, time-, and cost-intensive experimental setup of such studies.

There are two major methods to assess parasite viability, here defined as the capacity of a single parasite to re-establish a population. Both are based on the same initial step, the removal of the drug following the exposure time of interest. The first method (subsequently called “regrowth assay”) then closely monitors the drug-free parasites until they mature [11], reach a certain parasitemia [8,9], or show ability to re-infect fresh erythrocytes [12], so that the number of viable parasites present at the point of drug removal can be extrapolated assuming constant exponential growth. The second method (known as “parasite reduction ratio [PRR] assay” or “viability assay”) is based on limiting dilution of the drug-free parasites and subsequent regrowth. The PRR assay is independent of the assumption that parasites grow exponentially (whose growth rate might not be constant directly after drug treatment). Following limiting dilution and a cultivation period of several weeks, the number of viable parasites present at the point of drug removal can be deduced from the number of parasite-positive dilutions [6,10].

The first standardized version of the PRR assay was presented by Sanz et al. in 2012 [6]. This highly sensitive assay is used to study parasite viability following up to 120 h of drug treatment. From the resulting viability-time profile, valuable in vitro PD parameters such as the lag time, the PRR (log_10_ drop of viable parasites within 48 h), and the 99.9% parasite clearance time (PCT_99.9%_; time to kill 99.9% of parasites) can be deduced. With incubation periods of up to 28 days and subsequent manual data analysis, this assay is very time- and labor-intensive. Hence, the repeated calls for optimized viability assays [8,9,10] are not surprising. Here we report the development of the PRR assay version 2 (V2). This revised version comes with a shorter assay duration, optimized quality controls, and an objective, automated data analysis pipeline that systematically estimates the PRR, PCT_99.9%_ and the lag time and returns meaningful secondary parameters such as the maximal killing rate (E_max_) at the assayed drug concentration.

## 2. Results

### 2.1. Parasite Viability Vs. Standard Readouts

To demonstrate the value of measuring viable parasites when assessing antimalarial activity, we compared the PRR assay to two conventional in vitro drug screening methods—a microscopy- and a [^3^H] hypoxanthine incorporation-based growth inhibition assay. Four compounds covering diverse modes of action and antimalarial killing profiles served as references for this comparison (artemisinin, atovaquone, chloroquine, pyrimethamine) [6]. Their antimalarial activity was assessed following a 72 h drug pulse at ten times the 50% inhibitory concentration (IC_50_) (or 100 nM for atovaquone) by using either the PRR assay protocol presented in this work, a microscopic readout of the drug-free samples collected in that PRR assay directly after treatment, or an independent 72 h [^3^H] hypoxanthine incorporation-based growth inhibition assay.

**Figure 1:**
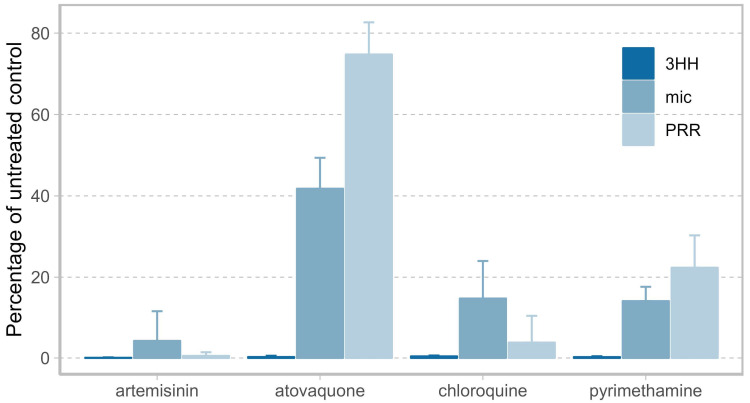
Comparison of drug activity after 72 h of drug exposure as determined by [^3^H] hypoxanthine incorporation (3HH, dark blue), microscopy (mic, blue), and by Parasite Reduction Ratio (PRR) assay (PRR, light blue) from three biological replicates with one, two, and four technical replicates for the microscopic-, [^3^H] hypoxanthine incorporation-, and the PRR assay, respectively. Resolution of drug activity is highest when measuring parasite viability using the PRR assay with compounds in order of decreasing activity: artemisinin, chloroquine, pyrimethamine, atovaquone. Artemisinin, chloroquine and pyrimethamine were examined at ten times the 50% inhibitory concentration (IC_50_), atovaquone at a concentration of 100 nM.

In the [^3^H] hypoxanthine incorporation-based growth inhibition assay, all compounds displayed similar antimalarial activities—irrespective of their mode of action—with less than 1% growth in comparison to the untreated control after 72 h of drug exposure (Figure 1). Although subjective, microscopic assessment of drug activity led to a more discernible ranking of the four drugs according to their antimalarial activity, with artemisinin being the most active, followed by chloroquine and pyrimethamine at equal levels, and finally by atovaquone, for which 40% of parasites were considered viable compared to baseline parasitemia. The resolution of drug activity was highest in the PRR assay, according to which drugs were ranked by their antimalarial activity in agreement with reported clinical observations [13] (in order of decreasing activity: artemisinin, chloroquine, pyrimethamine, atovaquone).

### 2.2. Optimizing the Protocol: The PRR Assay V2

**Figure 2:**
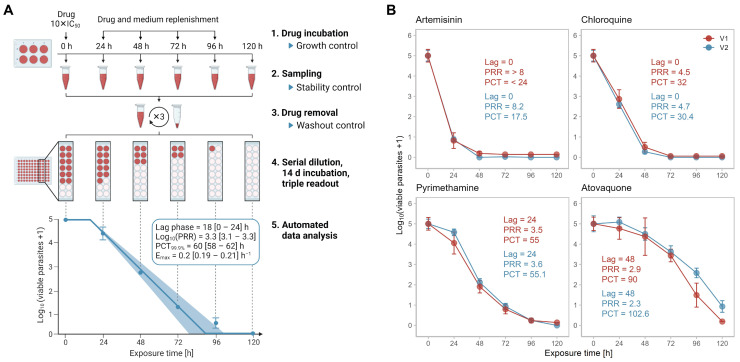
The in vitro PRR assay version 2 (V2). (**A**) Experimental setup. Cultures of *P. falciparum* strain NF54 are incubated with compound at 10× IC_50_, which was predetermined following 72 h of drug incubation. Drug is replenished every 24 h. At 0, 24, 48, 72, 96, and 120 h, a culture aliquot is taken and washed three times. Drug-free parasites are serially diluted and incubated in 96-well plates for 14 days in order to allow the viable parasites to resume growth to a measurable culture. Finally, the number of viable parasites present after washing can be extrapolated from the number of dilutions still yielding parasite-positive wells. An automated data analysis calculates pharmacodynamics (PD) parameters from the normalized raw data. In parallel, drug stability and washing efficiency are monitored with two bioassay-based quality controls and untreated parasite cultures serve as growth control. (**B**) Validation of the new experimental protocol with a shorter assay duration based on the reference compounds artemisinin, chloroquine, pyrimethamine and atovaquone. Killing profiles and PD parameters were obtained using the original PRR assay (V1, red) [6] or the PRR assay V2 (V2, blue) with a shorter assay duration. Drugs were tested at 10 × IC_50_ (or 100 nM for atovaquone in V2) in ≥three independent experiments; error bars represent standard error of the mean (SEM) of ≥four technical replicates; Lag = lag time (h), PRR = parasite reduction ratio, PCT = 99.9% parasite clearance time (h).

The optimizations incorporated in the PRR assay V2 concern the experimental protocol (reduced assay duration, comprehensive quality controls) as well as the downstream analysis of the acquired raw data (automated, algorithm-based data analysis) (Figure 2A).

As a first step, we assessed the general inter-laboratory reproducibility of the PRR assay as published by Sanz et al. [6] (Figure S1). For this, we tested three blinded compounds in *P. falciparum* strain NF54 and compared the results (upon disclosure) to those obtained with the original protocol. At this stage, we implemented only minor protocol changes, which were meant to align the assay conditions with those of the upstream IC_50_ assay and were not expected to affect the outcome. Overall, our results were in good agreement with those obtained using the original protocol.

#### 2.2.1. Reduced Assay Duration

The original PRR assay is time- and labour-intensive. It requires approximately one week of drug treatment followed by up to four weeks’ incubation of the drug-free cultures, with weekly replacements of the medium. The long incubation period has several drawbacks: it reduces the overall compound throughput and requires frequent culture handling, thereby increasing the risk of fungal- and cross-contaminations. Aiming to increase the efficiency of the assay and to reduce the risk of contaminations, we assessed the potential of a 14-day incubation period by means of the same four reference compounds (artemisinin, atovaquone, chloroquine, pyrimethamine) (Figure 2B). Because we expected the parasite densities to be low after only 14 days of incubation following drug removal, we implemented a triple readout to increase sensitivity: growth of drug-free parasites was verified by (i) [^3^H] hypoxanthine incorporation, (ii) growth-dependent discoloration of the filter mats on which the cultures were harvested, and (iii) growth-dependent discoloration of the medium observed during medium exchange. While (i) and (ii) served to detect low-density and regrowing cultures with high sensitivity, (iii) prevented false negatives caused by overgrown cultures that would no longer display a signal in (i) or (ii) at the time of readout.

We compared the combined results from at least three independent experiments to those generated by Sanz et al. [6] using the original PRR protocol (i.e., 28 days incubation). The resulting killing profiles were consistent with either protocol (Figure 2B), suggesting that 14 days of drug-free cultivation are sufficient to obtain detectable parasite numbers from an initial single parasite. Note that for atovaquone, a higher drug concentration is shown, since 10 × IC_50_ resulted in a plateau phase when using our protocol (Figure S2).

To further exclude the possibility that longer incubation periods would allow more parasites to grow up to detectable numbers (i.e., resulting in a higher number of parasite-positive wells), we also performed a direct comparison between a 14- and 21-day incubation period using pyrimethamine at 10 × IC_50_. For this, we incubated serially diluted parasite cultures derived from the same treated and washed sample for either 14 or 21 days. In three independent experiments, we found that the two incubation periods resulted in comparable killing profiles and PD parameters (Figure S3) indicating that 14 days of drug-free incubation indeed allow a single parasite to re-establish a measurable culture.

#### 2.2.2. Quality Control 1: Monitoring Undisturbed Parasite Growth

In every experiment, growth of untreated parasites was evaluated on the basis of 0 and 48 h samples (growth controls). Including additional samples would have been desirable for subsequent analysis, but was not possible due to potential stage effects (an initial parasite culture with a high proportion of ring stages will grow rather slowly within the first 24 h but much faster when undergoing schizogony towards the end of the 48 h) and limited erythrocyte supply (assuming a 10-fold increase in parasitemia every 48 h, an initial culture with 0.3% parasitemia would likely overgrow before reaching 96 h without the supplementation of fresh erythrocytes).

Initial experiments and simulations with 0 and 48 h samples revealed that PRR assay-derived parasite estimates of the untreated 48 h samples were not predictive of the actual parasite numbers; hence, the growth rate and the log_10_ parasite multiplication rate per 48 h (PMR) were also inaccurate (Figures S4–S6). The microscopically determined log_10_ PMR, in contrast, aligned with the literature [14] and our personal experience. Because the visual differentiation between viable and nonviable parasites is relatively simple and accurate in an untreated parasite culture at parasite densities within the range of the growth control of the PRR assay, we decided to use the microscopic readout to determine the experimental growth rate of the untreated control. The average *P. falciparum* NF54 growth rate as determined by microscopy was 0.048 [CI_95%_: 0.045, 0.052] h^−1^ at natural logarithm scale, which corresponds to a log_10_ PMR of 1.0 [0.94, 1.08] per 48 h.

#### 2.2.3. Quality Control 2 and 3: Monitoring Drug Stability and Removal

To ensure that (i) the drug and its concentration remained stable throughout the treatment period, and (ii) the drug had been fully removed before the parasites were incubated for regrowth, we established two bioassay-based quality controls (stability and washout control). For this, we collected supernatant before medium/drug exchange and during drug washout and assessed its antimalarial activity on naïve parasites.

In the drug stability assay, we assumed that the IC_50_ values for supernatants collected at the baseline and 24 h later (before drug replenishment) would be comparable for stable drugs, hence resulting in fold changes (IC_50, 24h_/IC_50, 0h_) close to one. Indeed, we found that three out of the four reference drugs were stable over a period of 24 h, as indicated by fold changes between 0.9 and 0.98 (Figure S7). With an IC_50_ fold change > 1.5, artemisinin was considered unstable, which is in agreement with published data [15]. To validate these bioassay-based results, we compared the obtained data to data derived from a liquid chromatography-mass spectrometry assay, where the concentrations in the supernatants were determined from two independent experiments. Both methods led to the same results (Figure S7).

The drug washout assay was based on the assumption that supernatants originating from insufficient drug washout would result in less parasite growth relative to a drug-free parasite control. We found that none of the supernatants collected during the last washing step inhibited parasite growth as determined by [^3^H] hypoxanthine incorporation suggesting that the drug washout was successful (data not shown).

#### 2.2.4. Automated and Objective Data Analysis

**Figure 3:**
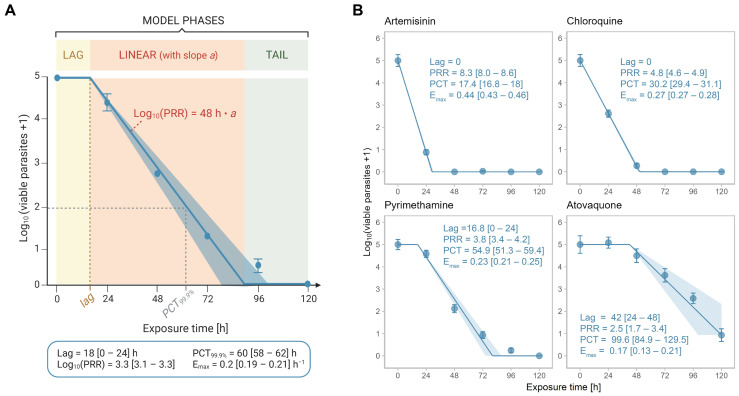
Automated data analysis method. (**A**) Schematic representation of the model. The model considers a lag, linear and tail phase. The lag time (lag) is chosen from a set of potential lags based on the best model fit and marks the end of the lag phase. The slope ‘a’ of the linear phase is used to determine the log_10_ (PRR) through multiplying by 48 h. The 99.9% parasite clearance time (PCT_99.9%_) is the time when 99.9% of the parasites are killed, i.e., when the log_10_ number of viable parasites is 2. Note that not all killing profiles possess a lag and/or a tail phase. (**B**) Killing profiles and PD parameters for the reference compounds artemisinin, chloroquine, pyrimethamine and atovaquone as determined by the new analysis method of PRR assay V2. Drugs were tested at 10 × IC_50_ (or 100 nM for atovaquone in V2) in ≥three independent experiments. Error bars represent SEM of ≥four technical replicates and ribbons represent the 95% confidence interval; Lag = lag time (h), PRR = parasite reduction ratio, PCT = 99.9% parasite clearance time (h), E_max_ = maximal killing rate (h^−1^); square brackets indicate the 24 h range (for lags) or the 95% confidence interval.

The slope of the linear phase of the killing profile is considered the most robust and relevant parameter for the assessment of drug efficacy [16,17]. Thus, the correct definition of the linear phase is essential for a precise estimation of drug efficacy. For compounds with a sigmoid viable parasite-time profile, such as pyrimethamine, this requires the distinction between lag, linear and tail phases (Figure 3A). Visual distinction between these segments is time-consuming and subjective and hence is a potential source of error. Thus, we developed an objective, automated approach to investigate the in vitro drug efficacy, which is based on the mathematical determination of lag, linear and tail phases of a viable parasite-time profile (Figure 3A).

Throughout the development process, several mathematical or semi-mathematical approaches have been developed and evaluated using the statistical software R (version 4.1.3) and amongst them, an adapted version of the parasite clearance calculator [17]. Most of these models, however, are overly complex and often estimate too many parameters from the limited number of measurements (six time points). The here-presented, best-performing model is a simple mathematical model from which only a single parameter (the slope of the linear phase) is estimated in the regression analysis, making it more determinable. The model assumes a nonlinear relationship between the duration (*t*) of exposure to a given drug and the number of viable parasites (*P_viable_*) at log_10_ scale. The underlying formula considers the lag phase, the linear phase, and the tail phase (a potential plateau at the end of the curve) of such a relationship (see Equation (2) in Section 4).

To determine the lag time, the model is run with various fixed lag times at incremental steps of 6 h. The model with the best fit (i.e., smallest residual standard deviation σ) is considered as final, unless one of four so-called ‘dominant rules’ applies (see Table S1 in Supplementary Materials). From this model, lag and slope of the log-linear phase are extracted and used to estimate the PRR, the E_max_, and the PCT_99.9%_. PRR and lag time are then used to assign a compound to one of five pharmacodynamic categories, which were formulated together with the Medicines for Malaria Venture (Table 1).

Based on the y-value of the tail phase, compounds can further be categorized as ‘complete’ (all parasites are dead after 120 h of treatment) or ‘partial killers’ (leaving > 0 parasites viable after 120 h of treatment). Finally, a warning message and visual signal are automatically created in a final written (.xlsx format) and graphic (.png format) summary if the standard deviation at a given exposure time is higher than one (corresponding to two times the average standard deviation of 93 data points from seven independent experiments), or if the simulation and the underlying measurements differ by more than the average standard deviation.

On the basis of 74 tested compounds, we performed a sensitivity analysis to determine the optimal length of increments that are used to define the duration of the lag phase based on the best model fit. For this, we investigated the changes in output variables (lag, PRR) when using 24 h (the actual assay resolution), 12 h, or 6 h lag phase increments instead of 3 h increments in the algorithm. We concluded that 6 h increments (¼ of the actual assay resolution) led to a satisfying trade-off between computation time and resolution, while being reasonably long to prevent over-interpretation of the limited number of measurements (Figure S8).

Re-analysing the data of the four reference drugs artemisinin, atovaquone, chloroquine and pyrimethamine at 10 × IC_50_ (or 100 nM for atovaquone) with the new algorithm, we found that the simulated killing profiles were consistent with the underlying raw data (i.e., averages from ≥three independent experiments) (Figure 3B). Similarly, there was a good agreement between the PD parameters from the old and new analysis methods with the pharmacodynamic ranking being conserved between the two methods.

**Figure 4:**
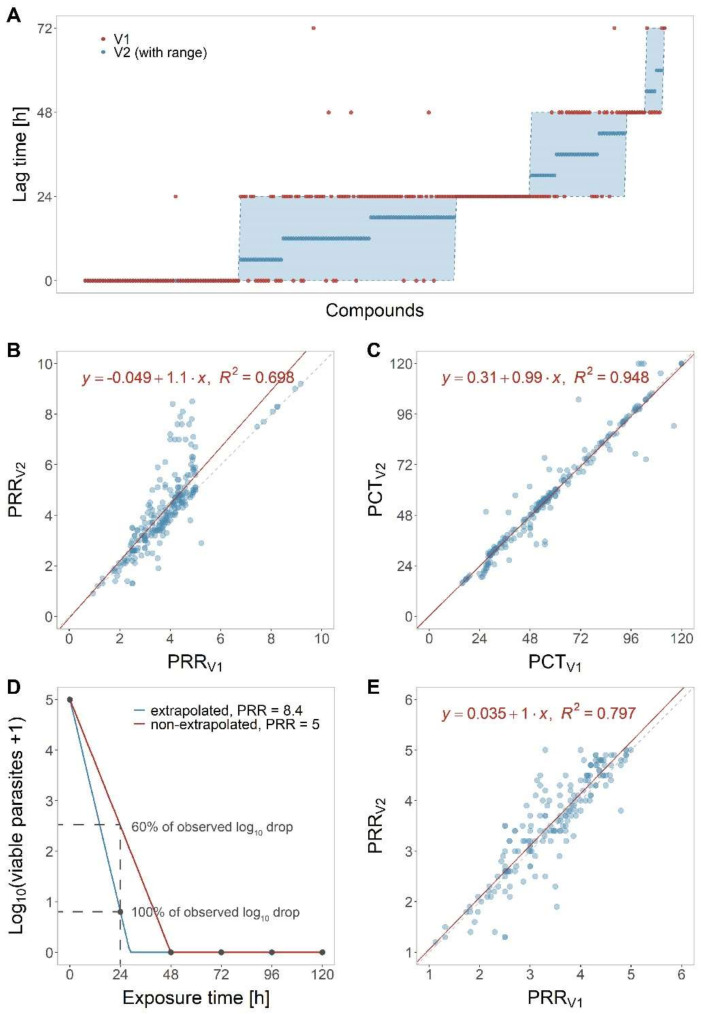
Validation of the new analysis method. PD parameter as obtained with PRR assay V1 or V2 analysis were compared for 232 molecules. (**A**) Comparison for lag time. (**B**) Comparison for PRR and (**C**) PCT_99.9%_: the dashed, grey line indicates identity of the two methods, the red line is the linear regression with the equation and R^2^ value displayed in the plot. (**D**) Demonstration of the effect on PRR values when extrapolating the slope between 0 and 24 h measurement to 48 h (blue) versus when not extrapolating (red). Not extrapolating results is underestimating the observed drop in parasites. (**E**) Linear regression for PRR when excluding all molecules with extrapolated PRR (PRR > 5) from the data set (n = 178).

We proceeded by validating the new analysis method on a data set comprising 232 compounds (of which 20% were slow-, 27% intermediate-, and 53% fast-acting) (Figure 4). The raw data of the compounds were extracted from a database of the Medicines for Malaria Venture and originated from different laboratories; they were analysed manually (according to PRR assay V1 [6]) and using the new algorithm presented here (PRR assay V2). Subsequently, the obtained PD parameters were compared between methods. The geometric mean fold errors (GMFEs) were 1.15 and 1.06 for PRR and PCT_99.9%_, respectively, and only 2.6% of the lag times obtained using the original method were outside the predicted range of those obtained using the new analysis method, thus indicating a good agreement between the two analysis methods (Figure 4A). For PRR or PCT_99.9%_, we also assessed the correlation between the two methods by a simple linear regression and found that they resulted in high R^2^ values and slopes close to identity (Figure 4B,C).

We further inspected compounds which displayed PRR and PCT_99.9%_ discrepancies across the two analysis methods (according to Bland–Altman analysis, fold-error > 1.2, and absolute difference of > 1 for PRR or > 12 h for PCT_99.9%_). Most of the PRR discrepancies were traceable to fast-acting compounds with artemisinin-like curve shapes, i.e., with drops in parasite numbers > 4 log_10_ units within 24 h of treatment. The PRR values obtained using the old analysis method (V1) were based on the linear stretch between 0 and 48 h; whereas, those analysed with the new analysis method (V2) were based on the linear stretch between 0 and 24 h extrapolated to 48 h (Figure 4D). In V2, we assume that the killing effect observed between 0 and 24 h continues until all parasites are killed. Although this means that the PRR exceeds the actual assay range (0 to 10^5^ parasites), this extrapolation step will result in expanding the assay’s range beyond the limitation of parasite numbers. Figure 4D illustrates the two approaches schematically: When comparing the curves generated with and without extrapolation at 24 h of drug exposure, we can appreciate that the extrapolated simulation (V2, blue) predicts a log_10_ reduction of approximately 4 after 24 h—similar to the underlying measurements. In the non-extrapolated simulation (V1, red), in contrast, the 24 h measurement (which results in a log_10_ reduction of approximately 2.5 at 24 h of drug exposure) is completely ignored, resulting in a gross underestimation of the observed drug effect by almost 40%. Removing compounds with an extrapolated PRR (i.e., compounds with PRR > 5) led to a better correlation between the two methods (Figure 4E), which indicates that extrapolating explains large parts of the observed discrepancies in PRR values.

#### 2.2.5. Inclusion of Growth Control Enables Calculation of E_max_

In the original PRR assay, all parasite samples (0–120 h samples) are collected from a single, drug-treated culture. After sampling, the remaining culture is centrifuged to replenish the drug and the medium. This means that the parasites collected at different time points had experienced varying rounds of centrifugation, i.e., parasites collected after 120 h of treatment experienced four rounds of centrifugation whereas those collected after 24 h experienced no centrifugation before drug removal. Consequently, a potential growth control (untreated culture sampled at 48 h), which would inform the experimental parasite growth rate in the absence of a drug, would be treated differently than most of the drug-treated samples.

In the PRR assay V2, the initial parasite culture is split into equal aliquots, one for each time point, before addition of the drug. In this setup, wells that are not destined for sampling at a given time point are not being resuspended, so that the medium and drug can be replenished without the need for centrifugation. Avoiding varying rounds of centrifugation between the samples (drug-treated samples and untreated controls) allows the same handling of all of them with minimal invasiveness. In addition, this allows an untreated growth control to be included, which is handled in the same way as the drug-exposed samples and can be used to determine the growth rate in every single experiment (the ‘experimental in vitro growth rate’). The average experimental growth rates (at natural logarithm scale) for *P. falciparum* strain 3D7 and NF54 were 0.041 (CI_95%_: 0.039, 0.043; n = 4) h^−1^ and 0.048 (CI_95%_: 0.045, 0.052; n = 7) h^−1^, respectively. The observed difference between the two *P. falciparum* strains was statistically significant (*p* < 0.05, Welch two-sample *t*-test).

With the experiment-specific growth rate at hand, the maximal hourly rate of parasite killing achieved by a given drug (E_max_, assuming that the rate of parasite killing has saturated at the given drug concentration) can be inferred by adding the rate of hypothetical growth (in the absence of drug) to the rate of parasite killing (in the presence of drug). The E_max_ is a key PD parameter required for dose prediction as it determines the drug duration necessary to eliminate all parasites from the patient’s blood [13,18]. Recently, the E_max_ derived from the in vitro PRR assay—together with data from the humanized mouse model—was shown to enable remarkably accurate predictions of effective doses in humans (unpublished results; manuscript in preparation).

A generic growth rate (0.048 h^−1^ at ln scale) has usually been used to calculate the in vitro E_max_. With the PRR assay V2, we now have a tool at hand to specifically determine the parasite growth rate in every single experiment. This will make it possible to better account for inter-experimental differences in parasite growth, resulting in more accurate E_max_ estimates. To investigate the difference between using a generic and an experimentally determined growth rate for E_max_ calculation, we deployed a simplified model that makes it possible to compare the time required to eliminate 10^12^ parasites (a common parasite burden in patients [13,19]) (Figure S9). As expected, the difference was highest for slow-acting compounds, where the time required to kill the parasites ranged from 213 to 238 h for the experimental growth rates versus 230 h for the generic growth rate. The higher the PRR of a compound, the smaller the relative weight of the growth rate. Overall, the experimentally determined growth rate results in more accurate estimates of E_max_ and hence a better understanding of a drug’s pharmacodynamics, an improved ranking of compounds’ in vitro efficacies and ultimately a better dose prediction.

## 3. Discussion

Parasite viability is being increasingly acknowledged as an effective measure of antimalarial drug activity—both in the preclinical and clinical contexts [7,8,9,20]. In contrast to conventional in vitro growth inhibition assays, parasite viability provides high-resolution information about a drug’s capacity to kill parasites and cure malaria.

The first attempts to assess parasite viability had aimed to overcome the limitations of microscopy and metabolism-based drug screening methods [10,11]. These studies were backed up by Sanz et al. in 2012 and, more recently, by mouse and human volunteer infection studies providing supportive evidence for the value of parasite viability as a readout to assess drug activity and monitor drug resistance [8,9].

In the present study, we found that parasite viability (as determined by the PRR assay) better discerned drugs according to their intrinsic antimalarial activity than microscopic- or isotopic-based growth inhibition assays. The difference was most pronounced for slow-acting compounds. Simply measuring the presence or absence of parasites independent of their viability (i.e., measuring ‘parasite clearance’) can lead to the underestimation of drug activity, as recently demonstrated for artesunate [8,9]. In fact, parasite clearance curves represent the combined effects of parasite killing and the removal of dead parasites by the spleen. This observation led to a call for the dissociation of parasite clearance and parasite killing [7]. On the other hand, a lack of parasite growth, as monitored e.g., by incorporation of [^3^H] hypoxanthine, does not necessarily imply the death of a parasite; it could also result from dormancy or cytostatic drug action. Misjudging growth inhibition for killing may result in overestimation of a drug’s antimalarial potential, as shown for atovaquone [6]. Such an overestimation might lead to subtherapeutic drug levels in the clinical context, hence facilitating the emergence of drug resistance. Therefore, we argue that although in the early discovery phase, growth inhibition remains the measure of choice of antimalarial activity, parasite viability needs to be assessed early on and used as a prioritization for further work.

In the PRR assay, parasite viability is defined as the capability of a drug-treated parasite to re-establish within a favorable environment, and given enough time after drug removal, a healthy parasite culture. Drug removal followed by a regrowth period makes it possible to discriminate cytostatic from cidal compounds at the relevant time points, and the limiting dilution technique allows estimation of the number of viable parasites with outstanding sensitivity [9]. The regrowth period must be sufficiently long to enable a single parasite to grow back to detectable numbers. The recommended time for this has been up to 28 days [6], resulting in five weeks for a single experiment and thus in a very low throughput. Using a faster multiplying *P. falciparum* strain and a triple readout, we demonstrated that 14 days of regrowth following drug removal are sufficient for a single parasite to establish a detectable population. This increases cost-effectiveness and throughput and reduces the risk of (cross-) contamination. A regrowth period of 14 days was also proven sufficient for the chloroquine-resistant *P. falciparum* strain W2 in a study conducted by Young and Rathod when monitoring parasite regrowth from a clonal culture using radiolabeled hypoxanthine [10]. However, this might be different for strains with slower growth rates, such as *P. falciparum* 3D7 used in the original PRR assay [6]. Strain-independent prerequisites for optimal growth are a healthy starting culture in fresh erythrocytes and a stable temperature and gas atmosphere combined with minimal handling outside these conditions. Therefore, we strongly recommend including an untreated growth control in each experiment.

Strain-specific differences in parasite growth might also explain the reduced atovaquone activity observed at 10 × IC_50_ in *P. falciparum* NF54 (Figure S3) as compared to the published values of *P. falciparum* 3D7 [6]. In fact, the killing curve generated here with 10 × IC_50_ atovaquone (corresponding to 6 nM, as determined by the authors of the present study) had a similar shape to the one published using 3 × IC_50_ (corresponding to 3 nM, as determined by the authors of the present study) in 3D7 [6] with both curves plateauing at 72 h (Figure S3). At higher concentrations, the plateau disappeared (Figure 3).

Following up on this, we identified the treatment conditions (treating and sampling from multiple wells instead of one large dish, Figure S10), differences in drug concentration, and the *P. falciparum* strain as potential causes for the observed discrepancy. We also investigated the possibility that the parasites might have acquired resistance after 120 h of exposure because atovaquone resistance is known to occur quite frequently [21,22]. However, no shift in IC_50_ was observed in parasites collected after 120 h of exposure (data not shown).

We have implemented two quality controls to monitor drug stability and drug removal, respectively. Incomplete drug removal leads to persisting drug pressure during the regrowth phase and hence to an overestimation of the drug’s antimalarial killing rate. This was the case with lipophilic synthetic ozonides that adhered to the plate surface during drug washout, highlighting the importance of an efficient washing protocol [23,24]. With this in mind, we deployed a bioassay-based washout control to test for complete drug removal. Another bioassay-based control was used to ensure that compound concentration remained stable over the course of drug treatment. This assay is of high pharmacological relevance, as it directly measures antiplasmodial activity of the tested compound and its potential metabolites. Moreover, mass spectrometry-based assessment of drug stability cannot be deployed for all types of compound, for example, if their inhibitory concentration falls below the technique’s limit of quantification or for other technical reasons; we were unable to quantify the concentration of a pyronaridine tetraphosphate using mass spectrometry, but this was readily achieved with the bioassay.

The publication on the original PRR assay V1 [6] provided the first standardized experimental viability testing protocol for the malaria field. Now we report the development of a complementary data analysis tool that computes relevant PD parameters in an automated and robust fashion. In contrast to other, more complex models we had investigated for the analysis of PRR data, the final model presented here is simple and robust, estimating a single parameter in the regression stage—the slope of the log-linear decay phase—from a limited set of measurements. Still, the limited number of measurements is a restriction of the assay, because it only yields a maximum of six time points to build the simulation on (for fast-acting compounds, often only two) given the 24 h sampling resolution. Working with clinical data, Flegg et al. showed that reducing the number of measurements leads to overestimation of the slope half-life mostly because of a delayed detection of the lag and tail phases [17], and White and Krishna recommended sampling intervals of four to six hours for accurate estimation of the parasite clearance rate [25]. In vitro, the sampling intervals are restricted by technical feasibility, e.g., the number of plates and cultures an experimenter can handle without overly extending the time for culture manipulation at room temperature and outside the preferred gas atmosphere. The 6 h incremental lag times chosen in the new PRR analysis algorithm makes it possible to increase the resolution without increasing the number of samples. Still, care should be taken considering that the actual measurements are based on 24 h intervals.

When comparing the PRR values generated with the old and new analysis method from the same underlying raw data, we found that the PRR values of fast-acting compounds were often higher when the new analysis method was applied. A likely explanation for this observation is that the new analysis tool extrapolates the killing effect from the first 24 h of drug action to 48 h (the duration of the parasite life cycle taken into account by the PRR) in very fast-acting compounds. On the one hand, this allows for a better ranking of these compounds: The old analysis method often resulted in a PRR value of five for both, a compound showing two consecutive log_10_ drops of 2.5 within the first 48 h of drug action (compound A), and a compound showing a log_10_ drop of 4 followed by a log_10_ drop of only one within the same time window (compound B). The new method, in contrast, calculated a PRR of five for compound A and—using extrapolation—a PRR of eight for compound B. This example also highlights that not extrapolating can result in underestimation of the observed drug effect, because a PRR of five implicates a 50% parasite reduction every 24 h, which is much less than the 80% reduction observed in the first 24 h of treatment with compound B. On the other hand, such a strong killing effect might not continue with the same intensity throughout the full 48 h if drug activity is stage-specific.

Thanks to the inclusion of an untreated growth control, the PRR assay V2 can estimate the specific E_max_ of a drug (in addition to lag time, PRR and PCT_99.9%_), taking into account the measured parasite growth in the absence of a drug. In the past, this was achieved by adding the PRR/h (slope of the log-linear decay phase) to a generic growth rate, i.e., assuming a 10-fold increase in parasitemia within 48 h in every experiment. However, the growth rate is influenced by the batch of erythrocytes, the *P. falciparum* strain as well as other, experiment-specific factors. Including a growth control in every experiment results in more accurate estimates of E_max_ and informs in case of suboptimal parasite growth.

Lastly, the limiting dilution technique—a crucial technical component of the PRR assay—finds a wide range of applicability outside of the malaria field, for example, to detect bacterial contaminations in the food industry or to determine virus-specific T lymphocytes [26,27]. The analytical component, namely, the algorithm proposed for the analysis of viable parasite-time data, might be useful for standardization of the analysis of non-malaria in vitro PD data, too. Adjustments to correct for diverging sampling frequency and other pathogens’ growth rates can be implemented readily into our R script, which has been made publicly available at https://github.com/annab3ll3/PRR-assay-V2.git (accessed on 13 January 2023).

## 4. Materials and Methods

### 4.1. Parasite Cultivation

The drug-sensitive *Plasmodium falciparum* strain NF54 (airport strain from The Netherlands) was provided by F. Hoffmann-La Roche Ltd. (Basel, Switzerland). Asexual blood stages were maintained at 37 °C and 93% N_2_, 4% CO_2_, and 3% O_2_ in humidified modular chambers and in accordance with Snyder et al. (2007) [28]. The culture medium consisted of RPMI 1640 (10.44 g/L) supplemented with HEPES (5.94 g/L), NaHCO_3_ (2.1 g/L), Neomycin (100 µg/mL), hypoxanthine (50 mg/L), and albuMAX^TM^ (5 g/L). The human erythrocytes were sourced ethically, and their research use was in accord with the terms of the informed consents under an IRB/EC approved protocol.

### 4.2. In Vitro [^3^H] Hypoxanthine Incorporation Assay

The in vitro antimalarial activity of compounds was measured in accordance with Snyder et al. (2007) [28]. In brief, parasites were exposed to a serial dilution of compound for a duration of 72 h. Addition of radiolabelled hypoxanthine after 48 h allowed the measurement of its incorporation into the parasites DNA, thus serving as a proxy of parasite growth [29]. Linear interpolation was used to determine IC_50_s [30]. IC_50_ values were in alignment with those published by Delves et al. [31].

### 4.3. Parasite Reduction Ratio Assay Version 2

In 6-well plates (Falcon #353046), unsynchronized *P. falciparum* parasites (strain NF54) with at least 64% of rings were incubated at 37 °C with either culture medium alone (serving as growth control) or together with fresh compound solution at 10 × IC_50_ if not stated otherwise (5 mL at 1.25% hematocrit and 0.3% parasitemia for each time point). Compound and/or culture medium were replenished every 24 h. Before the first treatment (0 h) and after 24, 48, 72, 96 and 120 h, 3 mL aliquots were sampled from the corresponding well (growth controls only at 0 and 48 h) and compound was removed by washing three times in 3 mL of culture medium (centrifugation: 2 min, 600× *g*). In 96-well plates (Sarstedt #83.3924), four technical replicates of each aliquot (eight for 0 h control) were serially diluted by factor four and incubated for up to 14 days at 37 °C. Once a week, culture medium was replenished and fresh erythrocytes at 1.5% hematocrit were provided. Thirteen days after serial dilution in 96-well plates, culture medium was replaced with 0.5 μCi of [^3^H] hypoxanthine in hypoxanthine-free medium, before freezing the plates 24 h later at −20 °C. Thawed plates were harvested with a Betaplate^TM^ cell harvester (Perkin Elmer, Waltham, MA, USA), which transferred the content of each well onto a glass fiber filter. The dried filters were inserted into a plastic foil with 3.5 mL of scintillation fluid and counted in a Betaplate^TM^ liquid scintillation counter (Perkin Elmer, Waltham, MA, USA). The results were recorded as counts per minute (cpm). Color changes of culture medium observed during the weekly medium replacements, and those observed on the dried glass fiber filters during the harvesting process served as additional, visual indicators of parasite growth. In every experiment, pyrimethamine and chloroquine were included as compound controls.

For each technical replicate of a sample, the number of viable parasites was extrapolated as follows:

(1) $$P_{viable\;}=\left\{\begin{array}{ccc} X^{n-1} & if & n\geq 1\\ \;0 & if & n=0 \end{array}\right.$$

where $P_{viable}$ represents the number of viable parasites, *X* the dilution factor used for serial dilution, and *n* the number of wells with detected parasite growth. The number of viable parasites at each sample time was log_10_ transformed and normalized using the initial sample (0 h), so that the 0 h samples of independent experiments always equaled 10^5^ parasites [6]. The normalized data (${\bar{P}}_{viable}$) were analyzed in R (version 4.0.3) [32] to derive the PD parameters. The growth rate was based on microscopic growth control data transformed to natural logarithm scale and corresponds to the slope of a linear model with a y-intercept fixed to 5 ·ln(10). Parasite viability following drug-treatment was estimated assuming a non-linear relationship between duration of exposure to a given compound (*t*) and ${\bar{P}}_{viable}$. The formula consists of a lag phase, a linear phase and a tail phase expressed as:

(2) $$f\left(t\right)=min(5,max\left(-a\;\cdot \;\left(t\;-\;lag\right)+5,\;minima\right))$$

where *f*(*t*) equals to $\log_{10}\left({\bar{P}}_{viable}+1\right)$, *a* represents the parasite decline rate, *lag* the lag time, and *minima* the tail taken as the minimum average $\log_{10}\left({\bar{P}}_{viable}+1\right)$ or 0 if the drop between 96 and 120 h was larger than the average standard deviation. The non-linear least squares function nls of R was used to estimate *a* and *minima* of the non-linear model (Equation (2)) for various fixed lag times (0, 6, 12, 18, […], 72 h). The best-fitting model (i.e., with smallest residual standard deviation σ) was considered as final, unless one of four so-called “dominant rules” applied (see Table S1 in the Supplementary Materials). From the final model, *lag* and *a* were extracted and used to estimate PRR (log_10_ drop of viable parasites within 48 h, Equation (3)), PCT_99.9%_ (time to kill 99.9% of parasites, Equation (4)), and *E_max_* (maximal killing rate assuming killing has saturated at 10 × IC_50_, Equation (5)) as follows:

(3) PRR=a · 48 h

(4) $${PCT}_{99.9\%}=\frac{3}{a}+lag$$

(5) $$E_{max}=a\;\cdot \;ln(10)+GR$$

where *a* is the parasite decline rate, *lag* the lag time, and *GR* the growth rate at natural logarithm scale. The fold error used to determine the geometric mean fold error (GMFE) in the method validation process was calculated as:

(6) $${Fold\;error=10}^{/log\frac{V2}{V1}/}$$

where *V1* and *V2* represent PD parameters (either PRR or PCT_99.9%_) as generated with the data analysis method of version 1 (Sanz et al. [6]), or version 2 of the PRR assay, respectively.

### 4.4. Quality Controls: Monitoring Drug Stability and -Washout

Throughout the whole treatment period, the compound was replaced every 24 h in order to maintain a stable drug concentration. To assess drug stability, the supernatant was collected 24 h after the first treatment. Compound solution collected at assay initiation (0 h) served as a reference. In a 96-well plate, two technical replicates of naïve *P. falciparum* parasites (strain NF54) were then exposed to a serial dilution of compound solution or supernatant at 1.25% hematocrit and 0.3% parasitemia and incubated at 37 °C and 93% N_2_, 4% CO_2_, and 3% O_2_. Inhibition of parasite growth was monitored using radiolabelled hypoxanthine added 48 h after initiation. Twenty-four hours later, plates were frozen at −20 °C before lysed cells were harvested as described in [28], and normalized counts were used to calculate the IC_50_. Drugs with IC_50_ fold changes (24 h/0 h) higher than 1.5 were considered instable.

To ensure that the drug removal had been efficient before the samples were serially diluted and incubated for regrowth in 96-well plates, supernatant was collected right before the last washing step and diluted in culture medium in accordance with the actual sample (i.e., to a two-fold higher concentration to account for the subsequent addition of naïve parasites). Two technical replicates of naïve *P. falciparum* parasites (strain NF54) were then exposed to a serial dilution of this supernatant at 1.25% hematocrit and 0.3% parasitemia and incubated at 37 °C and 93% N_2_, 4% CO_2_, and 3% O_2_. Inhibition of parasite growth was monitored using radiolabelled hypoxanthine added 48 h after initiation. Twenty-four hours later, plates were fully frozen at −20 °C before the lysed cells were harvested, as described above. Drug removal was considered successful when the growth of supernatant-treated parasites was comparable to that of the untreated controls, i.e., when the deviation of normalized counts was less than 20%.

## 5. Conclusions

Already in 1993, Young and Rathod emphasized the importance of viability as a measure to understand the mechanism of drug action, to aid the translation from in vitro to in vivo studies, and to monitor drug resistance [10]. The first standardized protocol for the quantification of viable parasites following drug treatment was presented by Sanz et al. in 2012. Now, we present an optimized version of the assay, the PRR assay V2. This assay comes with a shorter assay duration and with an objective, automated algorithm for robust data analysis. The latter makes it possible to determine not only the in vitro lag time, PRR and PCT_99.9%_, but also an estimate for the E_max_, the maximal killing effect of a drug, which takes the experimental growth rate into account and can be fed directly into pharmacodynamic/pharmacokinetic models, hence aiding dose prediction and drug lead optimization.

## Figures and Tables

**Table 1:** Definition of categories used for pharmacodynamic ranking for antimalarials.

Category	Criteria	Example
Fast without lag phase Fast with lag phase	PRR ≥ 4, lag = 0 h PRR ≥ 4, lag > 0 h	artemisinin, chloroquine
Intermediate without lag phase Intermediate with lag phase	3 ≤ PRR < 4, lag = 0 h 3 ≤ PRR < 4, lag > 0 h	pyrimethamine
Slow	PRR < 3 and/or lag ≥ 48 h	atovaquone

## Data Availability

Data is contained within the article and Supplementary Materials.

## Supplementary Materials

The following supporting information can be downloaded at: https://www.mdpi.com/article/10.3390/ph16020163/s1, Figure S1: Reproducibility of the PRR assay protocol, Figure S2: Atovaquone tested at various concentrations, Figure S3: Comparison of varying assay durations, Figure S4: In silico evidence for imprecision at high parasite densities, Figure S5: In vitro evidence for imprecision at high parasite densities, Figure S6: Microscopy is superior to the PRR assay when estimating high parasite numbers, Figure S7: Monitoring drug stability, Figure S8: Sensitivity analysis to define the lag phase increments, Figure S9: Generic vs. experimental growth rate, Figure S10: Comparison of treatment and sampling protocols; Table S1: Dominant rules for lag time determination.
