# Supplementary material for: The Parasite Reduction Ratio (PRR) Assay Version 2: Standardized Assessment of Plasmodium falciparum Viability after Antimalarial Treatment In Vitro
Source: Pharmaceuticals (Basel). 2023 Jan 23;16(2):163. doi: 10.3390/ph16020163 (PMC9959027; doi:10.3390/ph16020163)
Supplement: Supplementary file 1 [file pharmaceuticals-16-00163-s001.zip › pharmaceuticals-2142041-supplementary.pdf]

Supplementary Material

1. Figures

Figure S1: Reproducibility of the PRR Assay Protocol

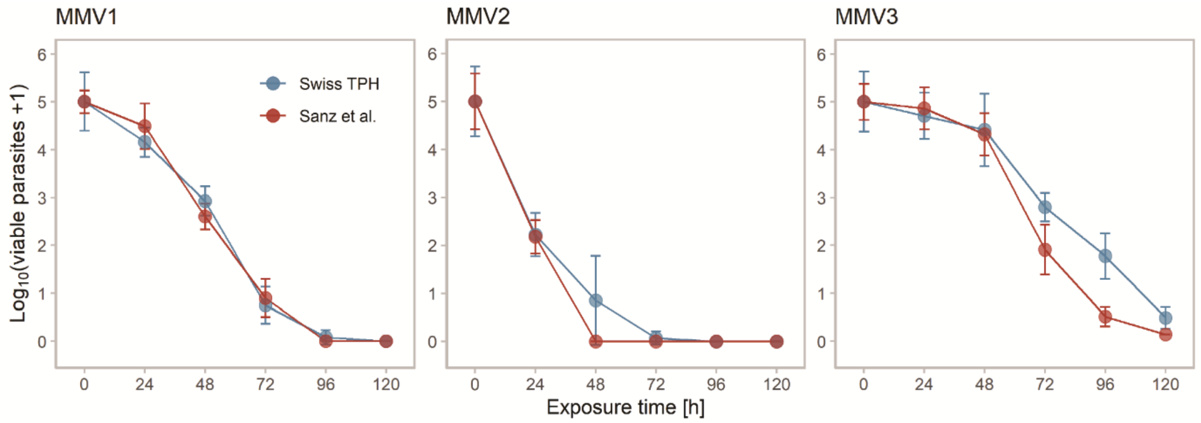

MMV1	Lag [h]	PRR	PCT [h]
Sanz et al.	24	3.6	57
Swiss TPH	24	3.4	57

MMV2	Lag [h]	PRR	PCT [h]
Sanz et al.	0	5.0	28
Swiss TPH	0	4.1	32

MMV3	Lag [h]	PRR	PCT [h]
Sanz et al.	48	3.8	75
Swiss TPH	48	2.6	91

Figure S1: Reproducibility of the PRR assay protocol. Spot testing of three blinded compounds indicates good inter-laboratory reproducibility of the PRR assay protocol. Killing profile and -parameters were generated at Swiss TPH (blue) or published by Sanz et al. [1] (red). For the blue curves the following changes were implemented: *P. falciparum* strain NF54 was used (instead of strain 3D7, a clone of NF54); 10 × IC_{50} drug concentrations were based on a 72 h [^{3}H] hypoxanthine incorporation assay (instead of 48 h); culture conditions were aligned with this [^{3}H] hypoxanthine incorporation assay; the washing protocol was extended; drug treatment and sampling was performed in and from independent wells instead of a single culture. All data were analysed using the original analysis method (Sanz et al.). Lag = lag time, PRR = log_{10} parasite reduction ratio, PCT = 99.9% parasite clearance time, error bars represent the standard deviation of four technical replicates.

Figure S2: Atovaquone Tested at Various Concentrations

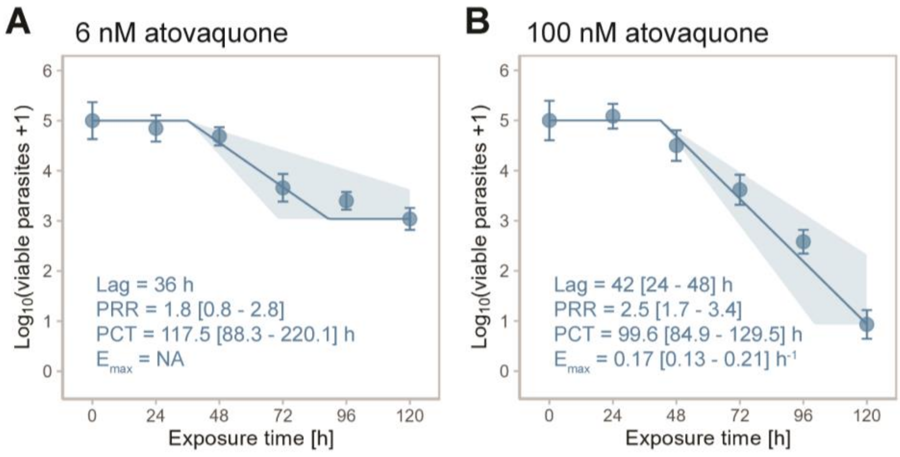

Figure S2: Atovaquone tested at various concentrations. PD profile and parameters for atovaquone at (A) 6 nM (10 × IC_{50}, as determined by the authors of the present study) and (B) 100 nM. Error bars represent standard error of the mean for n ≥ 3 biological replicates. These results were obtained with the PRR assay V2. Lag = lag time, PCT = 99.9% parasite clearance time, E_{max} = maximal killing rate.

Figure S3: Comparison of Varying Assay Durations

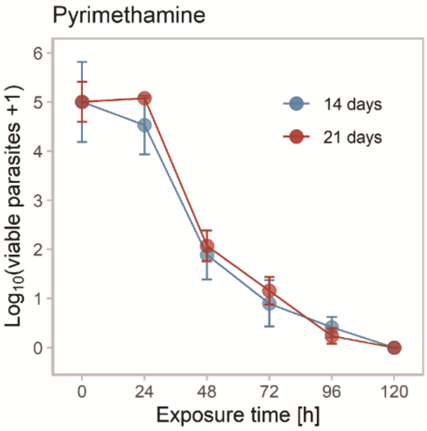

	Lag [h]	PRR	PCT [h]
14 days	24	3.6	54
21 days	24	3.9	57

Figure S3: Comparison of varying assay durations. Parasites were incubated with pyrimethamine at 10 × IC_{50} and then drug-free, serially diluted aliquots were incubated for either 14 (blue) or 21 days (red) in parallel. Graphs are representative for three independent experiments. Error bars indicate standard deviation of four technical replicates. All data were analysed using the original analysis method (Sanz et al.). Lag = lag time, PCT = 99.9% parasite clearance time, E_{max} = maximal killing rate.

Figure S4: In silico Evidence for Imprecision at High Parasite Densities

Early experiments revealed that parasite multiplication rates across 48 h (PMR_{48}) of untreated parasite samples were inaccurate when assessed using the PRR assay (using a 4-fold limiting dilution). To investigate whether the dilution factor used for limiting dilutions of the parasites had an impact on the accuracy of the untreated control estimates, we simulated the dilution process with either three- or four-fold dilution factor for 0 and 48 h untreated samples (growth controls) in the statistical software R. We assumed a parasite multiplication rate (PMR_{48}) of 1 to be normal, *i.e.* a 10-fold increase in parasitemia within 48 h. Consequently, an initial inoculum of 10^5 parasites (at 0 hours) was expected to increase to 10^6 parasites within 48 h. For both samples, we created virtual parasite populations of either 10^5 or 10^6 parasites coded as 1. Next, we simulated the three- or four-fold serial dilutions by taking 1/3 or 1/4 of these virtual parasite populations and mixed them with 2/3 (for three-fold dilution) or 3/4 (for four-fold dilution) of uninfected erythrocytes coded as 0. From the resulting mixed population of 10^5 or 10^6 0's and 1's, we randomly collected 1/3 or ¼ of the resulting population and mixed it with 2/3 or 3/4 of 0, thereby mimicking the serial dilution. We continued this process until we reached 15 dilutions.

Just as in real life, we then retrieved the number of dilutions with at least one virtual parasite (one 1) to calculate the number of parasites in the initial sample based on the dilution factor and determined the PMR_{48}. To reflect variability between real-life experiments, we repeated this virtual process 250 times and compared the distribution of these virtual parasite numbers to those obtained in 12 independent, real-life experiments with eight technical replicates per time point.

The data of the 0 h samples (10^5 parasites) revealed that both simulated dilution factors resulted in comparable distributions of parasite counts, which were in good alignment with the real-life data generated with a four-fold dilution (Figure S4A).

The data of the 48 h samples (10^6 parasites), however, clearly showed a wider distribution of the simulated parasite counts when using the four-fold dilution (Figure S4B). Besides, with $\log_{10}$ parasite counts up to 9.2, a subset of the real-world data exceeded the predicted range of the simulation, thereby suggesting that high parasite numbers cannot be accurately determined using the PRR assay protocol.

A 0 h (untreated, assuming 10^5 parasites)

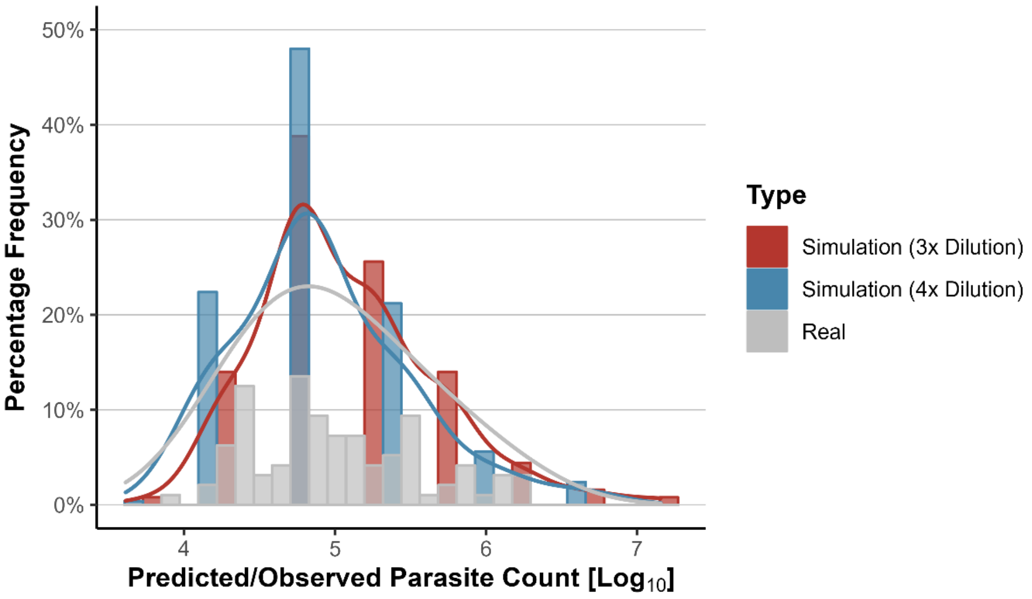

B 48 h (untreated, assuming 10^6 parasites with parasite multiplication rate of 1 within 48 h)

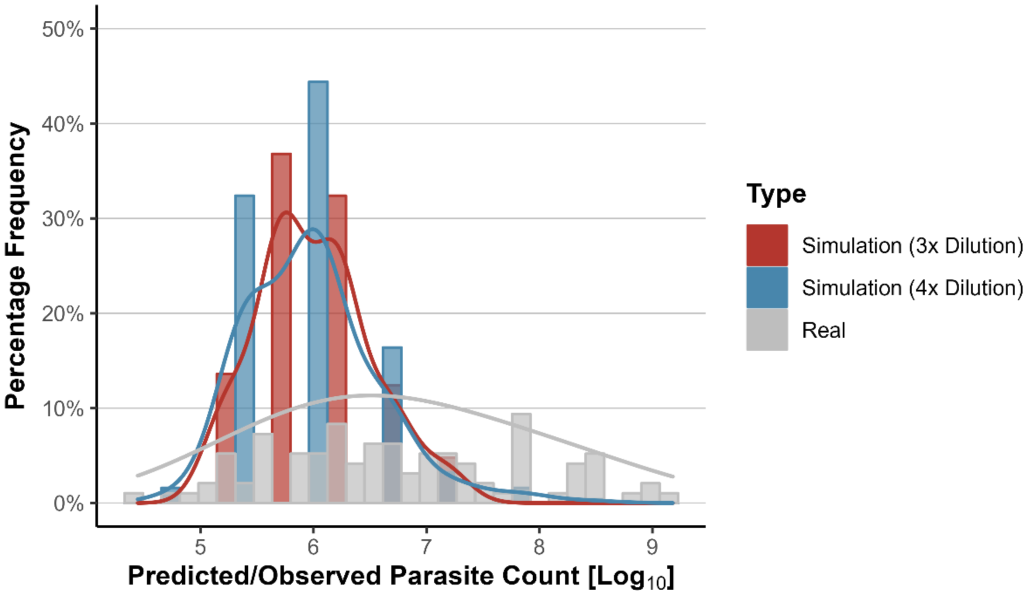

Figure S4: *In silico* evidence for imprecision at high parasite densities. Plots show distribution of predicted (virtual) and observed (real-life) parasite counts for (A) 0 h – and (B) 48 h growth controls. Predicted parasite counts were estimated using three-fold (red) or four-fold dilution (blue), real-life parasite counts (grey) are based on four-fold dilutions from 12 independent experiments with eight technical replicates per time point.

Figure S5: In vitro Evidence for Imprecision at High Parasite Densities

We hypothesized that the observed inaccuracy of the PRR assay is limited to high parasite numbers and does not affect the actual assay range (0-10^5 parasites). To test this hypothesis, we prepared a range of parasite dilutions from an initial sample of known, microscopically determined parasitemia. In accordance with the PRR assay protocol V2, each dilution was subjected to either three- or four-fold limiting dilution and consecutive incubation for 14 days. Figure S5 shows the comparison between expected and observed numbers of viable parasites for both dilution factors. The overall fit

between the observed and expected number of viable parasites was good (R^2 of 0.94 and 0.8 for three- and four-fold dilution, respectively). Importantly, both dilution factors are in good agreement throughout the actual assay range as also confirmed by the simulations (Figure S4). However, the distance from the 1:1 line is larger for dilutions containing ≥ 10^6 parasites (note that the estimates for the samples with the two highest parasite densities might be even higher if not restricted by the number of dilution steps performed, *i.e.* 7.2 ($log_{10}(3^{(16-1)}+1)$) and 9 ($log_{10}(4^{(16-1)}+1)$) for three- and four-fold dilutions, respectively.

A likely source of the observed inaccuracy at high parasite densities is the limiting dilution of drug-free parasites. Autoagglutination of parasitized cells or dilution errors both might result in overestimation of parasite numbers: The higher the parasite density, the more dilution steps are needed to reach the well with a single remaining parasite. More dilution steps, in turn, increase the impact of autoagglutination or the chance of a dilution error, *i.e.* transferring more than one third or one fourth of parasites to the next well.

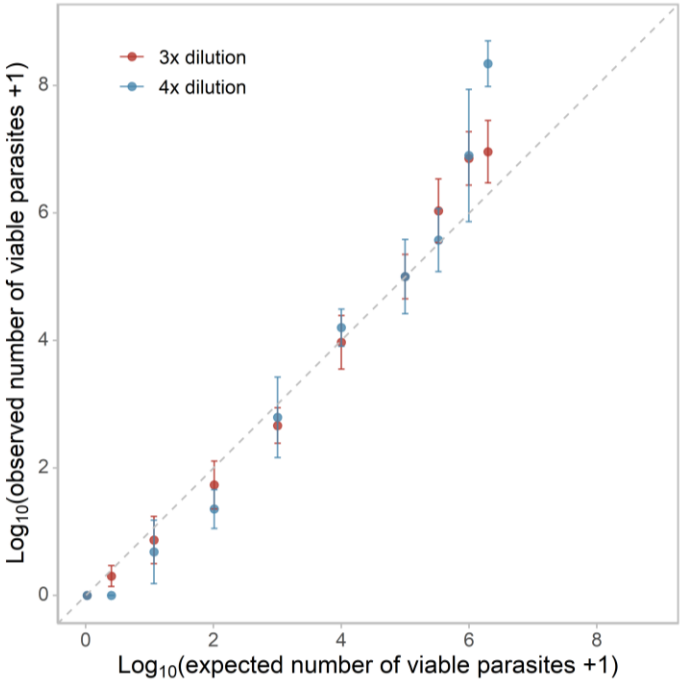

Figure S5: *In vitro* evidence for imprecision at high parasite densities. For 10 untreated samples containing between 0 and $10^{6.3}$ parasites, parasite densities were determined by three- (red) or four-fold limiting dilutions (blue) in a modified PRR assay and compared to the expected parasite densities as determined by microscopy. Error bars indicate the average standard deviation of two biological replicates each consisting of four technical replicates. The grey dashed line represents the identity line.

Besides, inaccuracy might also be linked to an increased risk of cross-contamination for samples with high initial parasitemia: When a large proportion of wells on a plate is parasitized, the chance of unintendedly transferring or spreading parasites to non-parasitized wells during the weekly medium replacements is high. When only few wells are parasitized, in contrast, the likelihood of spreading parasites to non-parasitized wells is much smaller.

Taken together, we show that parasite numbers exceeding the actual assay range can be inaccurate, hence also the estimate for the 48 h growth control sample, which usually contains around 10^6 parasites. This prevents accurate estimation of a growth rate through limiting dilution.

Figure S6: Microscopy is Superior to the PRR Assay When Estimating High Parasite Numbers

We then compared the log_{10} PMR (over 48 h) determined by microscopy and PRR assay with three- and four-fold dilution in three different parasite cultures. Again, the results confirmed that the PRR-derived log_{10} PMRs were misleading, e.g. a log_{10} PMR of ~2 (three-fold dilution) corresponding to a 100-fold increase in parasitemia (Figure S6). The microscopically determined log_{10} PMR of 1.08 ± 0.09, in contrast, aligned with the literature [2] and our personal experience. Since visual differentiation between viable and nonviable parasites is relatively straightforward in an untreated parasite culture at parasite densities within the growth control range, we decided to use the microscopic readout to determine the experimental growth rate for the untreated control.

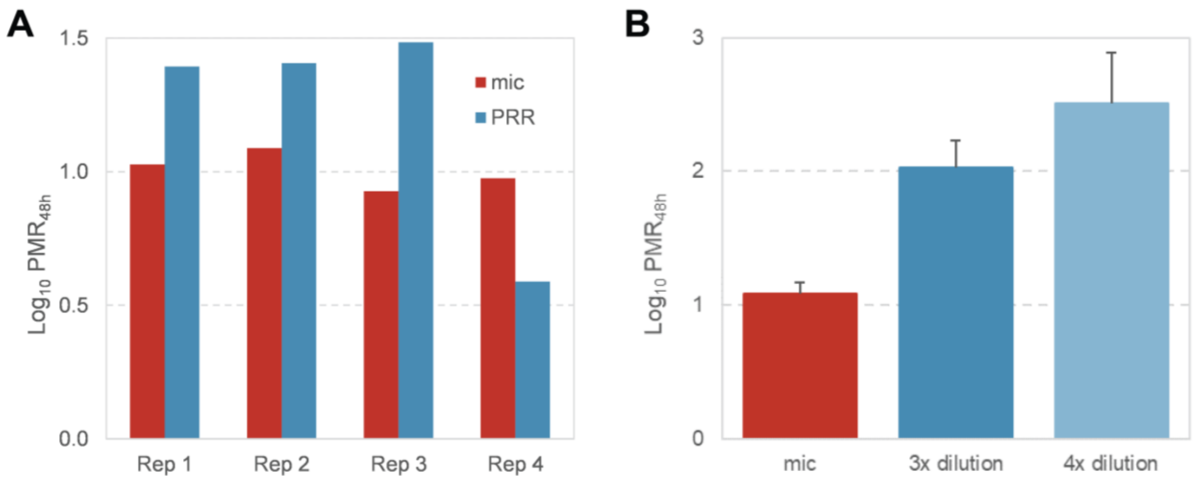

Figure S6: Microscopy is superior to the PRR assay when estimating high parasite numbers. (A) Microscopic (mic, red) and PRR assay PMR_{48} values (PRR, blue) for four independent experiments. (B) Microscopic PMR_{48} values (mic, red) in comparison to PRR assay PMR_{48} values using a three-fold (PRR, blue) or four-fold dilution factor (light blue) for limiting dilution. Error bars indicate standard deviation for three parasite cultures, PMR_{48} .is the 48 h parasite multiplication rate.

Figure S7: Monitoring Drug Stability

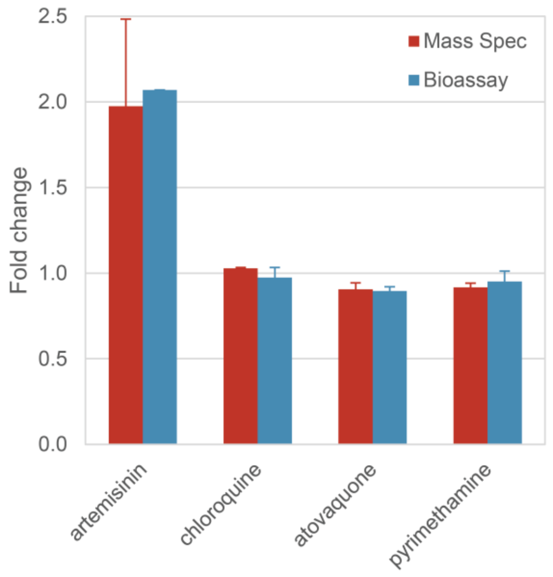

Figure S7: Monitoring drug stability. Comparison of drug stability as determined by liquid chromatography-mass spectrometry (Mass Spec, red) and the new bioassay (blue). Results are reported as mean fold change ± standard

deviation of drug concentration (mass spectrometry, 0 h vs. 24 h) or IC_{50} (bioassay, 24 h vs. 0 h) for two independent experiments.

Figure S8: Sensitivity Analysis to Define Lag Phase Increments

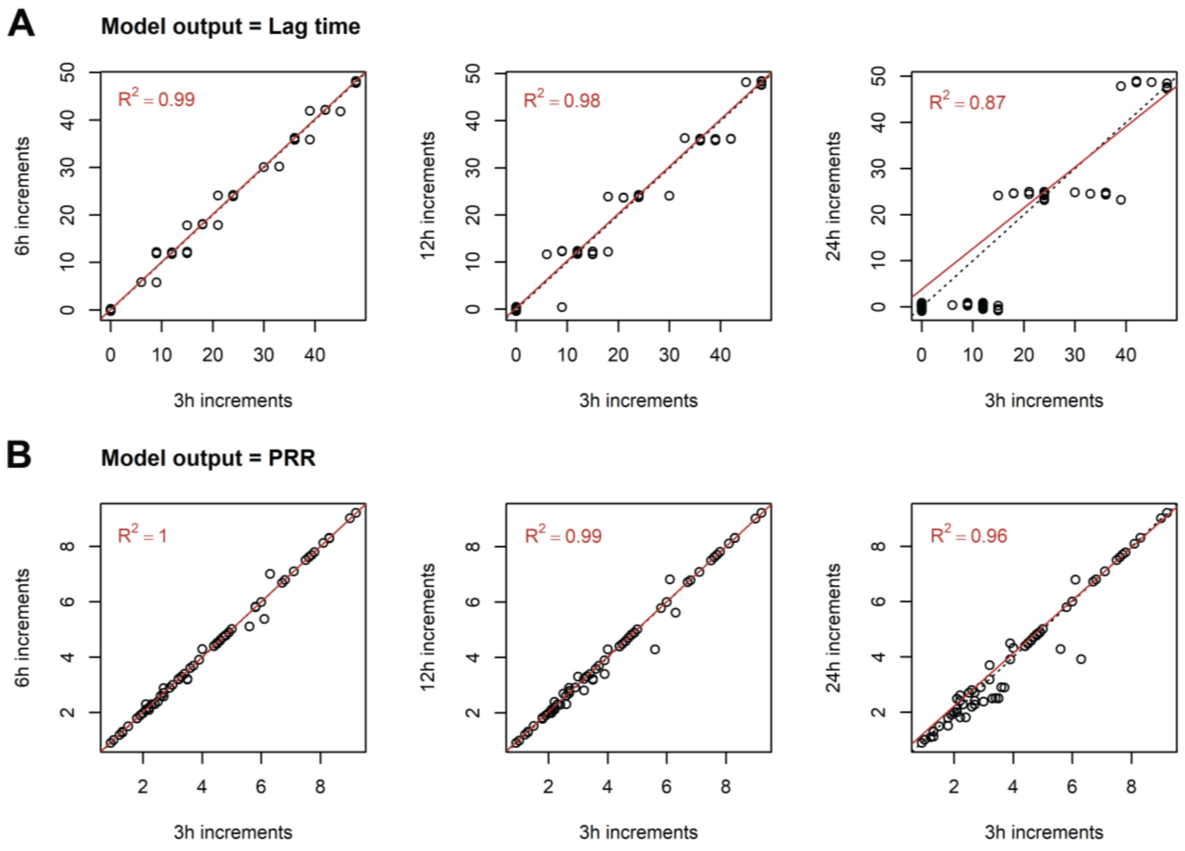

Figure S8: Sensitivity analysis to define the lag phase increments. Lag time (A) and PRR (B) of 74 compounds were determined by running the new data analysis algorithm with 6, 12, or 24 h lag increments and then compared to those determined with 3 h increments. The black dashed line represents the identity line, the red line is the regression line.

Figure S9: Generic vs. Experimental Growth Rate

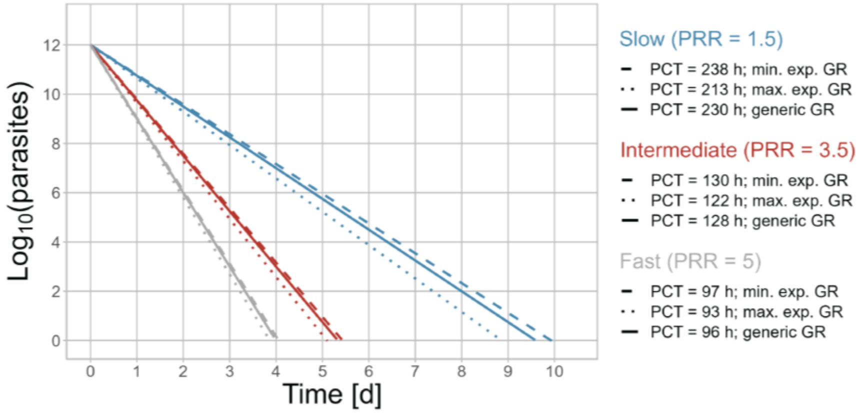

Figure S9: Generic *vs.* experimental growth rate (GR). For a slow-, intermediate-, and fast-acting compound, the E_{max} was calculated with the generic ($GR_{generic}$, 0.048 h^{-1}), the minimum and the maximum experimental GR (GR_{min}, 0.044 h^{-1}; GR_{max}, 0.058 h^{-1}). Subsequently, the time required to kill all parasites (PCT) was estimated for each growth rate and compound type from a simple linear model that assumes an initial parasite number of 10^{12} and a slope corresponding to the compound- and GR-specific E_{max}.

Figure S10: Comparison of Treatment and Sampling Protocols

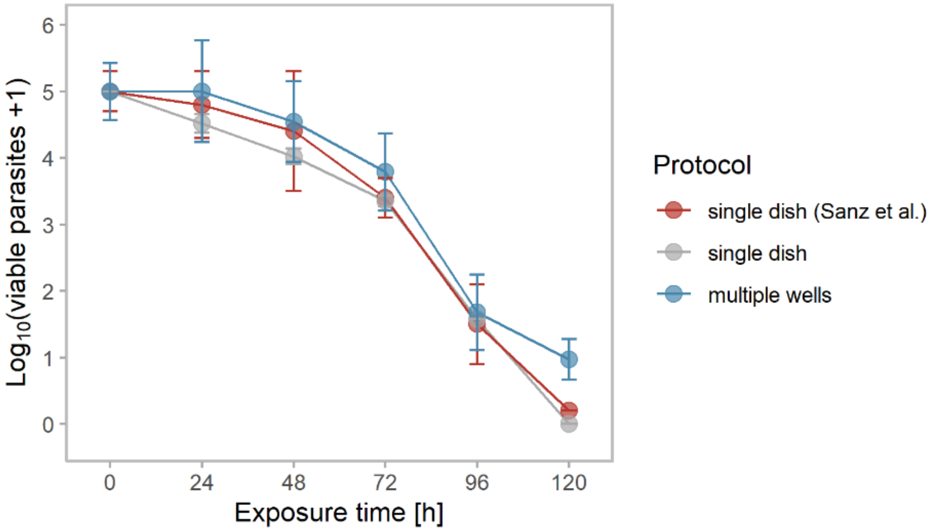

Figure S10: Comparison of treatment and sampling protocols. Cultures of *P. falciparum* strain 3D7 were exposed to 10 nM atovaquone. Treating and sampling parasites in and from multiple wells (blue, PRR assay V2) did not result in complete kill after 120 h of treatment. Treating and sampling in and from a single dish (grey, modified PRR assay V2 with treatment protocol by Sanz et al. [1]) resulted in a similar curve shape as published by Sanz et al. (red).

2. Tables

Table S1: Dominant Rules for Lag Phase Determination

The PRR assay V2 aims at introducing a more robust, objective and reproducible method for analyzing PRR assay data that returns the same outcome regardless of the user or analyzer. To combine human experience with computational rigor, we turned a number of rules that parasitologists generally apply using their "gut instinct" into an algorithm where such thinking is set into a stable and reproducible environment. This involves the introduction of four so-called "dominant rules", which may overrule the mathematical model in order to retain the model fit in a biologically relevant trajectory. In terms of lag times, for example, a regression model might at times ignore the fact that there was little to no change in parasite numbers within the first 24 hours of drug exposure. Instead, it assumes a lack of what is obviously a lag time in favor of a subtly better fit for later time points. Similarly, if a significant decrease of parasites is recorded at earlier time points, a regression model could regard this as a true lag time in favor of a slight improvement in fitting later observations. The biological importance of changes in initial time points however outweighs the importance of minute improvements in curve fitting at later time points. In summary, by means of the four dominant rules the PRR assay V2 analysis method appreciates the element of human experience instead of ignoring it in favor of objective functions and sigma values. It rather turns the entire process into one than can be performed consistently by tools that are free of human bias no matter the drug being analyzed.

Table S1: Dominant rules for lag time determination.

Rule no.	Description of dominant rule
1	If a lag time which is not part of the actual sampling times (0, 24, 48, 72, 96, 120 h) yields the best model fit (smallest σ value), but the σ value of a lag time which is part of the actual sampling times differs by ≤ 1% from the smallest σ value, the lag time which is part of the actual sampling times is considered as final lag time.
2	If the sum of half of the average standard deviation $\sigma_{average}$* and the average $log_{10}\ (P_{viable}+1)$ at an exposure time t_i is ≥ 5, then the lag time must be ≥ t_i.
3	If the sum of $\sigma_{average}$ and the average $log_{10}\ (P_{viable}+1)$ at an exposure time t_i with t_i > 24 h is ≥ 5, then the lag time must be ≥ $t_i - 12\ h$.
4	For t_i = 0 h: If $log_{10}\ (P_{viable}+1)$ decreases by ≥ 1.5 within t_i and $t_i + 24\ h$ of drug exposure, the final lag time must be < $t_i + 12\ h$. If $log_{10}\ (P_{viable}+1)$ decreases by ≥ 2 within t_i and $t_i + 24\ h$ of drug exposure, the final lag time is t_i.<br>For t_i > 0 h and lag time > t_i : If $log_{10}\ (P_{viable}+1)$ decreases by ≥ 1.5 within t_i and $t_i + 24\ h$ of drug exposure, the final lag time is $t_i + 6\ h$. If $log_{10}\ (P_{viable}+1)$ decreases by ≥ 2 within t_i and $t_i + 24\ h$ of drug exposure, the final lag time is t_i.

* Average standard deviation determined from 93 data points in seven independent experiments ($\sigma_{average}$ = 0.51).

3. References

1. Sanz, L.M., et al., *P. falciparum in vitro killing rates allow to discriminate between different antimalarial mode-of-action.* PLoS One, 2012. **7**(2): p. e30949.
2. Murray, L., et al., *Multiplication rate variation in the human malaria parasite Plasmodium falciparum.* Sci Rep, 2017. **7**(1): p. 6436.
